# Enhancing the Thermal Stability of Skyrmion in Magnetic Nanowires for Nanoscale Data Storage

**DOI:** 10.3390/nano14211763

**Published:** 2024-11-03

**Authors:** Mohammed Al Bahri, Mohammed Al Hinaai, Rayya Al Balushi, Salim Al-Kamiyani

**Affiliations:** Department of Basic and Applied Sciences, A’Sharqiyah University, P.O. Box 42, Ibra 400, Oman

**Keywords:** micromagnetic simulation, magnetic nanowire, skyrmion thermal stability

## Abstract

Magnetic skyrmion random switching and structural stability are critical limitations for storage data applications. Enhancing skyrmions’ magnetic properties could improve their thermal structural stability. Hence, micromagnetic calculation was carried out to explore the thermal nucleation and stability of skyrmions in magnetic nanodevices. Different magnetic properties such as uniaxial magnetic anisotropy energy (*Ku*), saturation magnetization (*Ms*) and Dzyaloshinskii—Moriya interaction (*DMI*) were used to assess the thermal stability of skyrmions in magnetic nanowires. For some values of *Ms* and *Ku*, the results verified that the skyrmion structure is stable at temperatures above 800 K, which is higher than room temperature. Additionally, manipulating the nanowire geometry was found to have a substantial effect on the thermal structural stability of the skyrmion in storage nanodevices. Increasing the nanowire dimensions, such as length or width, enhanced skyrmions’ structural stability against temperature fluctuations in nanodevices. Furthermore, the random nucleation of the skyrmions due to the device temperature was examined. It was shown that random skyrmion nucleation occurs at temperature values greater than 700 K. These findings make skyrmion devices suitable for storage applications.

## 1. Introduction

Researchers have shown significant interest in magnetic skyrmions within nanostructures due to their unique properties and potential applications in various advanced technologies [1,2]. Skyrmions are stable, topologically protected spin configurations that can exist in certain magnetic materials [3,4]. They are typically on the nanometer scale, making them highly relevant for nanotechnology applications and future electronic devices [5]. Additionally, skyrmions can be used to represent bits of data [6]. Due to their small size, they offer the possibility of extremely high-density data storage [7]. Driving magnetic skyrmions using a magnetic field is an area of active research due to its potential applications in data storage, logic devices and other spintronic technologies [8,9,10]. The manipulation of skyrmions with a magnetic field involves understanding their creation, movement and annihilation within magnetic materials [11,12,13]. Another way of driving magnetic skyrmions is through spin transfer torque (*STT*). *STT* is a phenomenon where the spin angular momentum from a spin-polarized current is transferred to the local magnetic moments in a material, causing them to move [14,15,16]. This mechanism can be used to manipulate magnetic skyrmions, which are topologically protected spin structures [17,18,19]. Driving skyrmions either by a magnetic field or spin transfer torque makes skyrmions a good candidate to have the potential to serve as magnetic bits in racetrack memory, offering advantages in terms of high density, speed and energy efficiency [20,21,22,23,24]. However, in magnetic nanowires, skyrmion racetrack memory faces stability challenges due to the used magnetic field, spin transfer torque, wire defects and device temperature [25,26,27]. Additionally, the skyrmion should be pinned at specific positions within the nanowire, such as at notches [28,29,30,31], constricted areas [32,33] and magnetic defects [34,35] to construct the magnetic nanowires as memory storage. Thus, understanding the effects of various parameters on the structural stability of skyrmions at the pinning sites is essential for applications. Most research has focused on examining the impact of pinning site geometry on skyrmion stability [36,37,38,39,40,41]. However, other influenced parameters, such as device temperature, have not been extensively considered as significant factors affecting skyrmion stability defects [42]. As skyrmion-based storage devices become smaller in nanoscale and more sensitive to temperature, the skyrmion’s structural stability due to the device temperature is essential in skyrmion storage data applications. While skyrmion structures have been found to be stable at low temperatures [43,44]. The novelty of this study lies in investigating the effect of magnetic properties on the thermal stability of skyrmions. Accordingly, this work focuses on the influence of device temperature on skyrmion structural stability in planar nanowires with nanoscale dimensions, as well as the effects of enhanced magnetic properties on skyrmion thermal structural stability. The thermal stability of the skyrmion was evaluated by modifying various magnetic properties, including magnetization (*Ms*) and uniaxial magnetic anisotropy energy (*Ku*). Additionally, the effect of nanowire geometry on the switching of skyrmions’ thermal stability was studied.

## 2. Theoretical Model

In this study, the calculations were performed by solving the Landau–Lifshitz–Gilbert (LLG) Equation (1):

(1) $$\frac{dm}{dt}=-\gamma m\;\times \;(H_{eff}+H_{th})+\alpha m\;\times \;\frac{dm}{dt}$$

In the equation, m represents the magnetization unit vector, γ denotes the electron gyromagnetic ratio, α stands for the dimensionless damping parameter, $H_{eff}\;$signifies the effective field and $H_{th}$ refers to the thermal field. The relationship between the device temperature (*T*) and the thermal field (***H****th*) is defined by Equation (2):

(2) $$\langle H_{th,i}\left(\vec{r,}t\right),H_{th,j}(\vec{\overset{\prime }{r}},\overset{\prime }{t}\rangle =\frac{2\alpha k_{B}T}{\gamma \mu_{0}M_{s}V}\delta_{ij}\delta (\vec{r}-\vec{\overset{\prime }{r}})\delta (t-\overset{\prime }{t})$$

**Figure 1:**
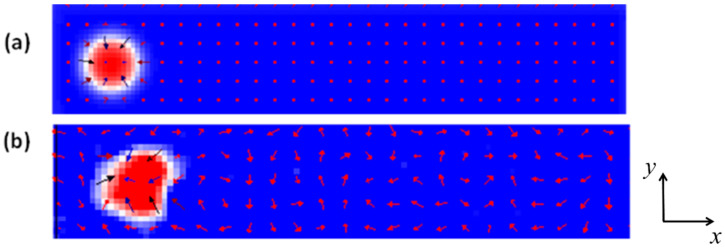
(**a**). The skyrmion (radius of 50 nm) at a device (200 nm × 40 nm × 3 nm) temperature of 0 K. The magnetization aligns in the negative z-direction in the initial state can be seen (blue) and in the positive z-direction (red). (**b**). The skyrmion under a temperature of 100 K in a nanowire with dimensions of (200 nm × 40 nm × 3 nm).

In this equation, T represents the device temperature, ${\;k}_{B}\;$stands for the Boltzmann constant, $\mu_{0}$ denotes the permeability and V indicates the computational cell volume. The simulations were performed using the Object Oriented Micromagnetic Framework (OOMMF) [45]. The initial state is shown as the blue region, where the magnetic moments are oriented towards the negative z-direction (*m_z_* = −1), while within the skyrmion (red region), the magnetization points upwards (*m_z_* = +1), as illustrated in Figure 1a, at device temperature of 0 K. Figure 1b illustrates the magnetization fluctuations of both the device and the skyrmion at a device temperature of 100 K. The study considers magnetic nanowires with dimensions of length (*L*), width (*w*) and thickness (*th*), specified as (200 nm × 40 nm × 3 nm). The radius of the skyrmion (*r*) is 50 nm.

The spin order and specific configuration that result in a stable skyrmion within a magnetic nanowire (Figure 1a) are determined by the total magnetic free energy density, which is a combination of several energy contributions: exchange energy, Dzyaloshinskii–Moriya interaction (DMI), anisotropy energy, Zeeman energy and magnetostatic energy. The total skyrmion magnetic free energy density can be expressed as follows:

(3) $$E=A{\left(\nabla M\right)}^{2}+D\left(M.\left(\nabla \;\times \;M\right)\right)+K_{u}{sin}^{2}\theta -\mu_{0}M.H-\frac{1}{2}\mu_{0}M.H_{d}$$

where A is the exchange stiffness constant, M is the magnetization vector, *D* is the DMI constant, *K_u_* represents the uniaxial anisotropy constant and$\;\mu_{0}$ is the permeability of free space [46,47,48]. However, as the device temperature increases, the spins experience more random orientation, leading to higher spin disorder and skyrmion collapse, as shown in Figure 1b [49].

## 3. Results and Discussion

The steady motion of the skyrmion is described by the Thiele equation:

(4) $$G\;\times \;\left(v^{\left(s\right)}-v^{\left(d\right)}\right)+D\left(\beta v^{\left(s\right)}-{\alpha v}^{\left(d\right)}\right)+\nabla V(r)$$

where $v^{\left(d\right)}$ is the drift velocity, $v^{\left(s\right)}$ is the velocity induced by the spin-polarized current, G is the gyromagnetic coupling vector, D represents the dissipative force and V(r) is the confining potential induced by the sample edges [50]. The gyro-term originates from the Berry phase and causes a moving skyrmion to be deflected perpendicular to its direction of motion. However, the stability of skyrmion motion in magnetic nanowires is a crucial aspect for potential applications in spintronics and memory storage devices. Skyrmions, which are topologically stable spin textures, exhibit robust dynamic behavior when subjected to external forces such as electric currents, magnetic fields and thermal gradients. The stability of their motion is influenced by several factors, including material properties, geometric constraints and external perturbations.

**Figure 2:**
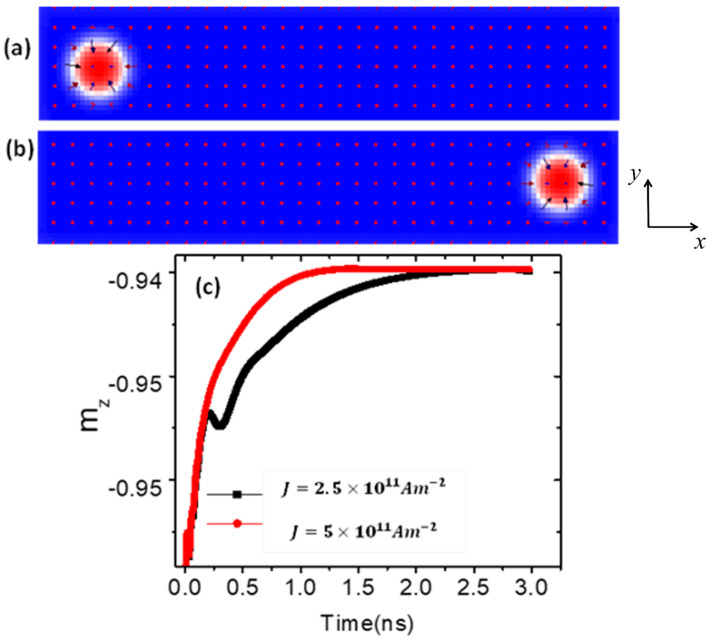
(**a**) The skyrmion at 0 K with a radius of 50 nm and magnetization aligned along the positive *z*-axis (red) at t = 0 ns. (**b**) The skyrmion under a temperature of 0 K reaches the end of a nanowire with dimensions (200 nm × 40 nm × 3 nm). The magnetic properties are *Ku* = 0.5  ×  10^5^ Jm^−3^ and $Ms=1.0\;\times \;10^{6}\;Am^{-1}.$ (**c**) Normalized *m_z_* versus time in a magnetic nanowire under two values of current density.

Thus, the main objective of this study is to enhance skyrmion thermal stability and nucleation in magnetic nanodevices based on device temperature. The structural stability of skyrmions is first investigated by driving them to the end of a nanowire at different current densities under a simulated temperature of 0 K. It was observed that at 0 K, no change in the skyrmion diameter occurred as it moved to the end of the nanowire, as shown in Figure 2a,b.

**Figure 3:**
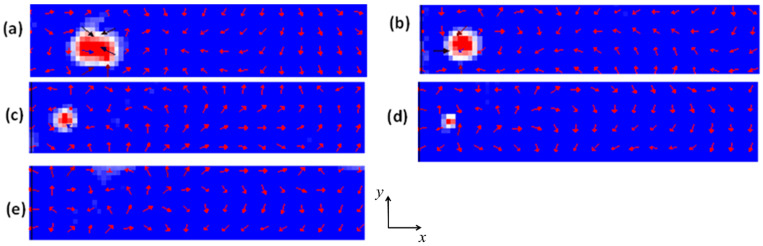
Skyrmion in a device with dimensions of 200 nm × 40 nm × 3 nm at a temperature of 150 K after (**a**) 0.65 ns, (**b**) 2.27 ns, (**c**) 2.50 ns, (**d**) 3.1 ns. (**e**) The skyrmion completely annihilates at a device temperature of 150 K after 4 ns. The magnetic properties are *Ku* = 0.5  ×  10^5^ Jm^−3^ and $Ms=1.0\;\times \;10^{6}\;Am^{-1}$.

To further confirm the skyrmions’ structural stability during their dynamics in the magnetic nanowire, a normalized magnetization component in the z-direction (*m_z_*) was plotted as a function of time for two current density values, as shown in Figure 2c. However, as shown in Figure 3a, once the device temperature reached 150 K, the magnetization fluctuated slightly along the *z*-axis. Due to these magnetization fluctuations, the skyrmion lost its stability, gradually shrinking in size over time until it completely annihilated after 4 ns, as shown in Figure 3b–e.

**Figure 4:**
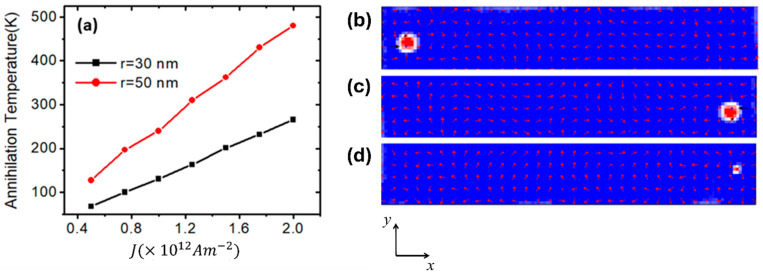
(**a**) Skyrmion annihilation temperature versus current density for skyrmion radii of 30 nm and 50 nm. (**b**) Skyrmion motion to the right in magnetic nanowire under a specific value of current density. (**c**) Skyrmion propagation under $J=1.2\;\times \;10^{12}\;Am^{-2}$ at a device’s temperature of 100 K. (**d**) Skyrmion propagation under $J=1.2\;\times \;10^{12}\;Am^{-2}$ at a device’s temperature of 150 K. The magnetic properties are $Ms=1.5\;\;\times \;\;10^{6}\;Am^{-1}$ and *Ku* = 0.5 × 10^5^ Jm^−3^. The nanowire dimensions are 200 nm × 40 nm × 3 nm.

Investigating skyrmion propagation in nanowires is essential for developing skyrmion-based devices with applications in information storage, computing and spintronic technologies. Hence, the thermal stability of skyrmions during their propagation in nanodevices was examined under different current density values. This study examines two skyrmions with radii of 30 nm and 50 nm across various device temperatures. It was found that the relationship between skyrmion annihilation temperature and driven current density is nearly linear as shown in Figure 4a. At certain device temperature and current density values, the skyrmion reached the end nanowire with high structural stability. For example, Figure 4b,c show the propagation of the skyrmion (*r* = 30 nm) under $J=1.2\;\times \;10^{12}\;Am^{-2}$ at a device temperature of 100 K. However, when the device temperature was raised to 150 K, the skyrmion, driven by the same value of current density, lost its stability and annihilated before reaching the edge of the nanowire, as shown in Figure 4d.

**Figure 5:**
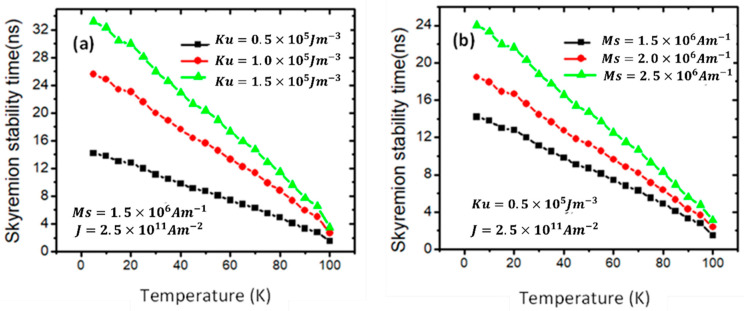
Graph of skyrmion stability time (*r* = 50 nm) versus device temperature in nanowires (200 nm × 40 nm × 3 nm) for three values of (**a**) *Ku* and (**b**) *Ms*.

There are different ways to enhance the thermal stability of the skyrmion, either by developing magnetic properties such as *Ms* and *Ku* or by manipulating the skyrmion and device dimensions. Therefore, the stability time of the skyrmion was investigated as a function of device temperature for three values of *Ku* (Figure 5a) and *Ms* (Figure 5b). Figure 5a shows that the stability time of the skyrmion decreases as the device temperature increases. For example, with a *Ku* value of $0.5\;\times \;10^{5}\;Jm^{-3}$, the skyrmion was stable for 5 ns under a device’s temperature of 80 K, while at a device’s temperature of 20 K, it remained for 13 ns. The stability time shows a similar trend with *Ku*, as the skyrmions’ stability time increases with increasing *Ku*. For instance, at a device temperature of 50 K, the graph indicates that the skyrmion stability time was around 9 ns with *Ku* of $0.5\;\times \;10^{5}\;Jm^{-3}$, while it was 20 ns with a *Ku* value of $1.5\;\times \;10^{5}\;Jm^{-3}$.

Similarly, the skyrmion stability time increased as *Ms* was increased, as shown in Figure 5b. For example, at a device temperature of 30 K, the stability time was 11 ns with$\;Ms=1.5\;\times \;10^{6}\;Am^{-1}$, whereas it increased to 19 ns with $Ms=2.5\;\times \;10^{6}\;Am^{-1}$. The increase in the skyrmion’s structural stability by increasing *Ms* is attributed to an increase in exchange energy [31].

**Figure 6:**
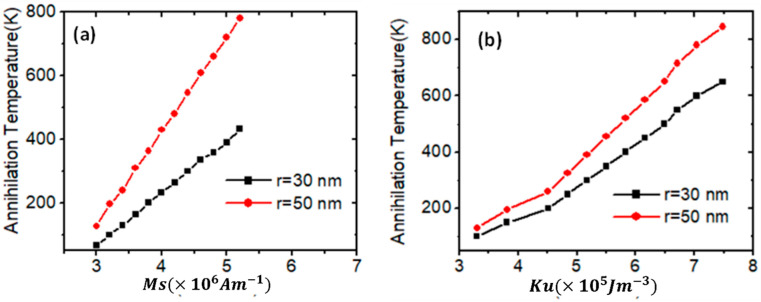
Skyrmion annihilation temperature versus *Ms* for skyrmion radii of 30 nm and 50 nm: (**a**) and *Ku* (**b**). The current density used is $J=2.5\;\;\times \;\;10^{11}\;Am^{-2}$, and the nanowire dimensions are 200 nm × 40 nm × 3 nm.

To enhance the structural stability time of the skyrmion at temperatures higher than room temperature, the skyrmion annihilation temperature was investigated by developing magnetic properties. Figure 6a shows the skyrmion annihilation temperature versus *Ms* for two values of skyrmion radii (*r*). For *Ms* values greater than 3.5 × 10^6^ $Am^{-1}$, the skyrmion was found to be more stable at room temperature and above. It was observed that for certain *Ms* values, skyrmions remain stable at temperatures up to 750 K with a skyrmion radius of 50 nm and up to 480 K with a radius of 30 nm, which is significant for storage applications. Similar results were found when increasing *Ku*; for *Ku* values greater than $6.5\;\times \;10^{5}\;Jm^{-3}$, skyrmions remained stable at device temperature up to 400 K and 800 K with r = 30 nm and 50 nm, as shown in Figure 6b.

**Figure 7:**
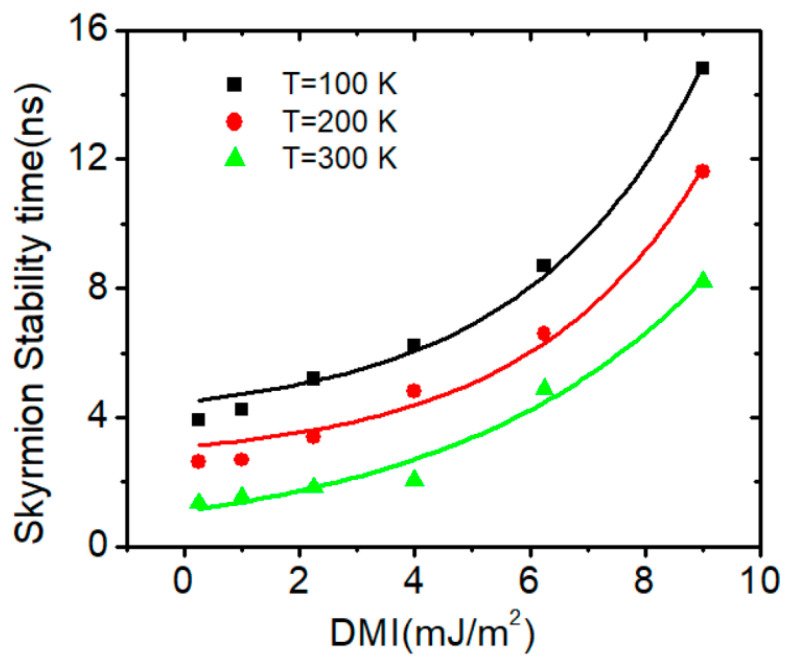
Plots of skyrmion stability time versus DMI for device temperatures of 100 K, 200 K and 300 K under *J* = 2.5 × 10^11^ Am^−2^, *Ku* = 0.5 × 10^5^ Jm^−3^ and $Ms=1.5\;\;\times \;10^{6}\;Am^{-1}$. The skyrmion (r = 50 nm) is in a nanowire with 200 nm × 40 nm × 3 nm.

The effect of *DMI* interaction energy on the skyrmion’s thermal stability was investigated. It was found that the skyrmion structure becomes more stable against device temperature by increasing *DMI* energy. For example, at a device temperature of 100 K, the skyrmion stability time increased from 4 ns with a *DMI* value of 0.2 mJm^−2^ to around 14 ns with a *DMI* value of 9 mJm^−2^. Similar improvements in the skyrmions’ structural stability were observed at device temperatures of 200 K and 300 K, at which *DMI* values increased. Figure 7 shows the skyrmion stability time as a function of *DMI* for three different device temperatures.

**Figure 8:**
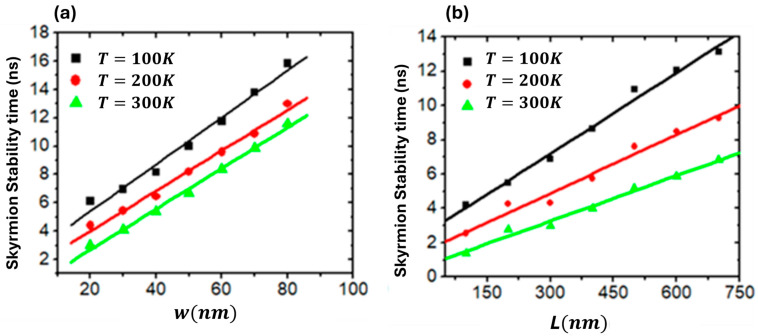
Plots of skyrmion (*r* = 50 nm) stability time in nanowires versus (**a**) device width (*w*) and (**b**) device length (*L*) for a range of temperatures (100 K, 200 K and 300 K). The current density used is *J* = 2.5 × 10^11^ Am^−2^, $Ms=1.5\;\;\times \;10^{6}\;Am^{-1}$ and *Ku* = 0.5 × 10^5^ Jm^−3^.

Furthermore, skyrmion stability time against device temperature can be adjusted by manipulating device dimensions, such as length (*L*), width (*w*) and thickness (*th*). This study examined the relationship between skyrmion thermal stability time and *w* over a range of temperatures (100 K, 200 K and 300 K). The results showed that the relation between thermal stability time and *w* is nearly linear, as illustrated in Figure 8a. Skyrmion stability improves with increasing device width. Thermal skyrmion stability is also influenced by the nanowire length, an inherent aspect of device geometry. Therefore, nanodevices with different *L* values were analyzed, demonstrating significant stability, as shown in Figure 8b. These results indicate that the skyrmion thermal stability time increases linearly with device length.

**Figure 9:**
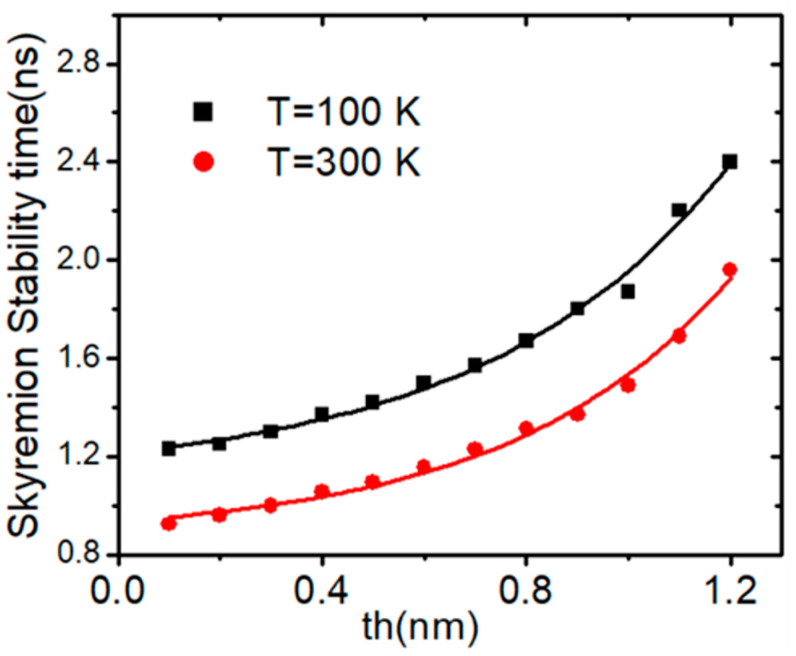
Plots of skyrmion stability time in nanowires versus (a) device thickness (th) for two device temperatures (100 K and 200 K). Nanowire dimensions are 200 nm × 40 nm × 3 nm.

The effect of device thickness (*th*) on skyrmion thermal stability was examined at two temperatures (100 K and 300 K). It was found that the skyrmion stability time exponentially increased for both temperatures, as shown in Figure 9a, which shows the dependence of skyrmion stability time (t) against device thickness (*th*) for the range temperatures of the device. This dependence can be described by an exponential relationship:

(5) $$t=t_{0}+Ae^{th/\rho }$$

Here, (th) represents the device thickness in the *x*-direction, while ρ and **A** are fitting parameters. The values of ***ρ*** and ***A*** at both temperature values are shown in Table 1.

**Figure 10:**
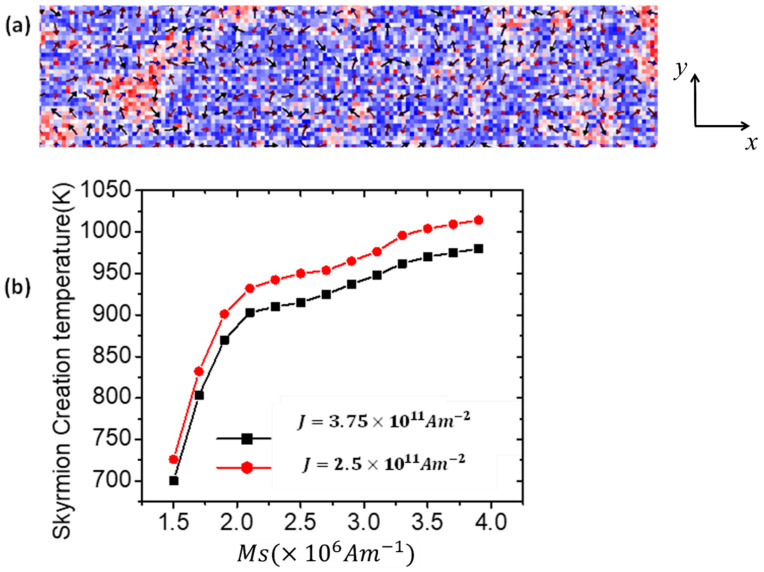
(**a**) Random thermal creation of a skyrmion in a nanowire (200 nm × 40 nm × 3 nm) under a device temperature of 700 K. (**b**) Plot of skyrmion creation temperature against *Ms* for two current density values.

Another part of this study focused on random skyrmion switching under varying device temperatures. Device failure can be attributed to random skyrmion generation caused by temperature changes. Accordingly, this study examined how device temperature affects skyrmion generation in nanoscale nanowires., as shown in Figure 10a. The thermal nucleation of skyrmions was investigated by varying saturation magnetization (*Ms*) under different values of current densities, as shown in Figure 10b. In Figure 10b, the nucleation temperature of a skyrmion increased sharply with an increasing *Ms* value in the range of 1.5 × 10^6^ Am^−1^ ≤ *Ms* ≤ 3.5 × 10^6^ Am^−1^, while remaining nearly constant for *Ms* values exceeding 3.5 × 10^6^ Am^−1^. The graph indicates that random skyrmion thermal switching occurs at temperature above 700 K suggesting that skyrmions are promising candidates for storage applications at elevated temperatures.

In addition, decreasing nanowire dimensions, such as the width, decreases skyrmion thermal switching due to an increase in shape anisotropy energy. It was found that no skyrmion thermal nucleation occurred at device temperatures greater than 900 K for *Ms* values of $\geq 2.5\;\times \;10^{6}\;Am^{-1}$.

## 4. Conclusions

To conclude, this study examined how magnetic properties and device dimensions can be used to control the thermal structural stability of skyrmion. By improving magnetic properties of magnetic materials such as *Ms* and *Ku*, it is possible to control the thermal structural stability the skyrmion in magnetic nanowires. The results showed that skyrmion thermal stability increased by increasing *Ms* and *Ku* values. At temperatures around 800 K, skyrmions were found to be stable with certain magnetic material properties. making the skyrmion structure more stable above room temperature. Consequently, this has the potential to extend the lifetime of device memory. Additionally, manipulating nanowire dimensions such as length, width and thickness contributed to increased skyrmion thermal structural stability. For some nanodevice dimensions, the skyrmion structure remained stable up to 900 K. Simulations also indicated that increasing device thickness reduces skyrmion magnetization switching. These findings may support the further development of skyrmion thermal stability in nanodevices.

## Figures and Tables

**Table 1:** Parameters for fitting derived from the exponential growth function of skyrmion time stability time against *th* for two device temperatures, as illustrated in Figure 9.

T (K)	$t_{0}$ (s)	*A* (s)	ρ (nm)
100	1.11 ± 0.05	0.10 ± 0.03	9.48 ± 0.96
300	0.87 ± 0.04	0.07 ± 0.02	8.63±0.82

## Data Availability

Data are contained within the article.
